# Double Salts of Carbolic Acid

**Published:** 1870-10

**Authors:** 


Double Salts of Carbolic Acid. — The difficulties in the administration of carbolic acid have peen overcome in the discovery and employment of salts of the sulpho-carbolic acid ($C_6H_6S\ O_4$) with the alkaline, earthy and metallic bases. The first compound salt, the sulpho-carbolate of potash, was obtained by Crooks. Dr. Sansom has produced in addition the following salts, all having the character of true double salts, and possessing brilliancy and decidedly crystalline forms: sulpho-carbolate of sodium, of potassium, of ammonium, of magnesium, of zinc, of copper, and of iron. An inquiry instituted with a view of determining the relative efficiency of the various salts in staying fermentative action, established the following results;—1, the sodium salt; 2, magnesium; 3, potassium; 4, ammonium. It has been shown, from experiments upon the lower animals, as well as from the results of administration to the human subject, that the following is an outline of the plan of action of the sulpho-carbolates: They are absorbed with great rapidity, exert no toxic effect (the human subject readily taking drachm doses every four hours), are decomposed in the system into—*a*, carbolic acid which, traversing the system, is exhaled by the breath; *b*, sulphate of soda, which permeates the tissues after death. It is shown that an influence, enabling the body to resist putrefaction, has been exerted; the urine passed also resists decomposition. Prolonged courses of sulpho-carbolate of sodium, given for two months to phthisical patients, show that the drug could be administered not only with impunity, but with considerable advantage. Of 35 cases, 13 greatly improved; 15 considerably improved; and 9 cases gained in weight an average of 2½ pounds.—*Med. Times and Gazette, Half-Yearly Com.*

